# Cell Entry of Animal Coronaviruses

**DOI:** 10.3390/v13101977

**Published:** 2021-10-01

**Authors:** Yang-Ran Cheng, Xinglin Li, Xuesen Zhao, Hanxin Lin

**Affiliations:** 1 Department of Pathology and Laboratory Medicine, Western University, 1151 Richmond Street, London, ON N6A 3K7, Canada; acheng2002@gmail.com; 2 Institute of Infectious Disease, Beijing Ditan Hospital, Capital Medical University, Beijing 100015, China; lixl0514@163.com; 3 Beijing Key Laboratory of Emerging Infectious Disease, Beijing 100015, China; 4 Molecular Genetics Laboratory, London Health Sciences Centre, 800 Commissioners Road East, London, ON N6A 5W9, Canada

**Keywords:** animal coronaviruses, virus entry, cell binding, membrane fusion, spike protein, receptor, attachment factor, proteolytic cleavage, host restriction factor

## Abstract

Coronaviruses (CoVs) are a group of enveloped positive-sense RNA viruses and can cause deadly diseases in animals and humans. Cell entry is the first and essential step of successful virus infection and can be divided into two ongoing steps: cell binding and membrane fusion. Over the past two decades, stimulated by the global outbreak of SARS-CoV and pandemic of SARS-CoV-2, numerous efforts have been made in the CoV research. As a result, significant progress has been achieved in our understanding of the cell entry process. Here, we review the current knowledge of this essential process, including the viral and host components involved in cell binding and membrane fusion, molecular mechanisms of their interactions, and the sites of virus entry. We highlight the recent findings of host restriction factors that inhibit CoVs entry. This knowledge not only enhances our understanding of the cell entry process, pathogenesis, tissue tropism, host range, and interspecies-transmission of CoVs but also provides a theoretical basis to design effective preventive and therapeutic strategies to control CoVs infection.

## 1. Introduction

Coronaviruses (CoVs) are a group of enveloped, single-stranded positive-sense RNA viruses that cause diseases in the respiratory, gastrointestinal, hepatic, and central nervous systems [1]. The genome size ranges from 26.4 to 31.7 kb, making CoVs the largest RNA viruses known thus far. CoVs are associated with an extremely wide range of vertebrate hosts. So far, 45 CoV species have been identified from avian and mammalian species including human, domestic, wildlife, terrestrial, aerial, and marine animals. These CoVs fall into four genera, *Alpha-*, *Beta-*, *Gamma-*, *and Deltacoronavirus*, under the *Orthocoronavirinae* subfamily, *Coronaviridae* family, *Cornidovirineae* suborder, and *Nidovirale* order (https://talk.ictvonline.org/taxonomy/, accessed on 9 September 2021).

CoVs have long been known as economically important pathogens of livestock, poultry, and pet animals. For example, *infectious bronchitis virus* (IBV) was considered as the number one cause of infectious disease-related economic loss in the UK poultry industry [2]. *Porcine transmissible gasteroenteritis virus* (TGEV) causes nearly 100% fatality in piglets [3].

**Figure 1:**
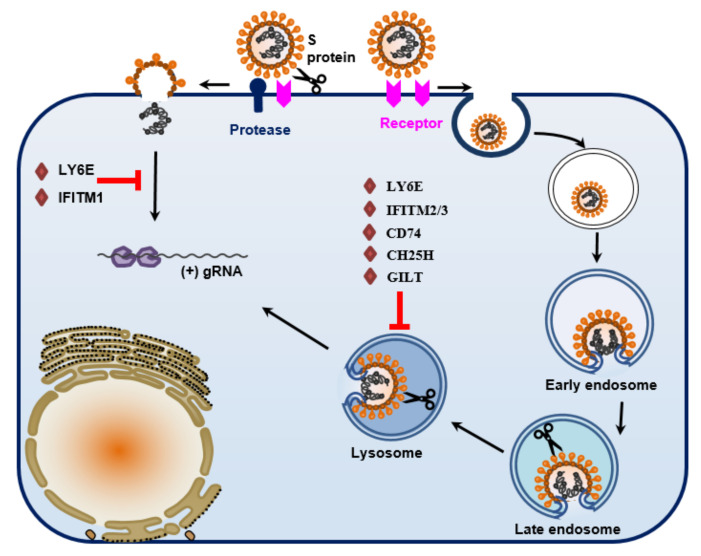
Schematic model for cell entry process of coronaviruses. The virus entry process is initiated by cell binding, which is mediated by the interaction between the viral spike (S) protein and cellular receptor and/or attachment factor, followed by membrane fusion and release of the viral genome into the cytoplasm for replication. Membrane fusion occurs at the plasma membrane (early entry) in a pH-independent manner or in endosome/lysosome compartments (late entry), in a pH-dependent manner, or both. Proteolytic cleavage of the S protein is a critical priming step for membrane fusion. Cleavage can occur at the plasma membrane by TMPRSS2 or trypsin or in the endosome/lysosome by cathepsin. Membrane fusion occurring at the cell surface could be inhibited by interferon-inducible host proteins LY6E and IFITM1. Membrane fusion in the endosome/lysosome can be restricted by several other interferon-inducible proteins, including LY6E, IFITM2, IFITM3, CD74, CH25H, and GILT.

Cell entry is the first and essential step of virus infection (Figure 1). Although it is an ongoing process, conceptually, it can be divided into two steps: cell binding and membrane fusion. The former is involved in the binding of viral spike (S) protein to cell surface components, i.e., cellular receptors and/or attachment factors. Following cell binding, the S protein undergoes a conformational change, followed by membrane fusion between virus particles and host cells, and subsequent release of virus genome into the cytoplasm. Membrane fusion occurs either at the plasma membrane in a pH-independent manner, or in the endosome in a pH-dependent manner. Many factors affect virus entry, tissue tropism, persistence, virulence, and host range. These include the specific receptor usage, binding to attachment factors, susceptibility to protease cleavage, acid-induced conformational changes, and host restriction factors [4,5,6].

In this review, we primarily focus on the cell entry of coronaviruses infecting economically significant animals and pets, including porcine, chicken, bovine, canine, and feline coronaviruses, but will also cover the research advances in human coronaviruses (HCoVs), particularly in the discovery of host restriction factors for entry.

## 2. Overview of Coronaviral S Proteins

As the outermost component of the virion, the S protein is the major viral determinant for cell entry, host range, tissue, and cell tropisms. This is well exemplified by two reciprocal studies showing that *murine hepatitis virus* (MHV) recombined with *feline infectious peritonitis virus* (FIPV) S protein acquired the ability to infect feline cells and simultaneously lost the ability to infect mouse cells [7], while FIPV recombined with MHV S protein no longer infected feline cells but was able to infect mouse cells [8].

**Figure 2:**
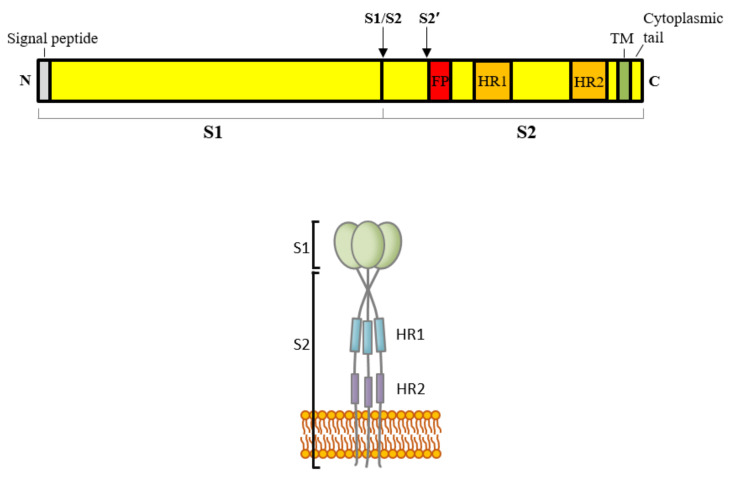
Schematic diagram of the domain architecture of coronaviral spike (S) proteins. Upper panel: domain architecture. Lower panel: a model of S trimer where the S1 forms a globular head and the S2 forms a narrow stalk. FP: fusion peptide. HR1: heptad repeat 1. HR2: heptad repeat 2. TM: transmembrane domain. S1/S2 and S2′ are two types of proteolytic cleavage sites. The S1 domain is required for receptor binding, while the S2 domain mediates membrane fusion.

The S proteins of coronaviruses, varying from 1160 to 1452 amino acids in length, are a type I transmembrane protein that contains a signal peptide, a long N-terminal ectodomain, a transmembrane (TM) domain, and a short C-terminal cytoplasmic tail (Figure 2). The signal peptide directs the nascent S polypeptide to the endoplasmic reticulum (ER), where the signal peptide is cleaved and monomeric S precursors are synthesized. The precursors are heavily glycosylated to yield 150–200 kDa matured monomers, which are further oligomerized into homotrimers in the ER [9].

CoV S proteins are a class I fusion protein, similar to the S proteins of orthomyxoviruses, retroviruses, paramyxoviruses, and filoviruses. Class I fusion proteins have several common features: (i) they are type I transmembrane glycoproteins; (ii) the mature protein is a homotrimer; (iii) they can be cleaved into two noncovalently associated subunits, S1 and S2, by host proteases; (iv) the amino-terminal S1 subunit forms the globular head and is responsible for receptor binding, while the carboxyl-terminal S2 subunit forms a narrow stalk and mediates membrane fusion (Figure 2); and (v) the S2 domain harbors a fusion peptide and two heptad repeats (HRs) that forms a characteristic six-helix bundle structure during membrane fusion [6].

In addition to the role in receptor binding, the S1 domain is also the major determinant to elicit the production of neutralizing antibody. Some anti-S1 antibodies could block receptor binding and protect animals from infection [6,10]. Many coronaviruses have variations in the S1 domain that result in immune response escape. Minor variations as few as one amino acid could change receptor usage, tissue tropism, and virulence [11,12]. Compared to the S2 domain, which is considerably conserved, the S1 domains are diverse even between different strains, and this genetic diversity accounts for the different host range, receptor specificity, virus tropism, antigenicity, and virulence [4].

## 3. Cell Binding

### 3.1. Cellular Glycoprotein Receptor

Cellular receptors have been identified for several CoVs (Table 1). The MHV receptor, carcinoembryonic antigen-related cell adhesion molecule 1 (CEACAM1; also called CD66a), is the first entry receptor identified for CoVs [13,14,15]. Full-length CEACAM1 contains four Ig-like domains, but some isoforms have one or two domains missing and thus exhibit less S-binding affinity [13]. Glycosylation of CEACAM1 is required for receptor function [16]. The MHV S-binding domain and neutralizing antibody epitope are overlapping in the N-terminal 108aa of CEACAM1 [17]. Further characterization identified six critical S-binding residues (aa 38–43) on an exposed loop [18,19,20]. Crystal structure and mutagenesis analyses showed that a total of 17 residues in the domain D1 of CEACM1 interact with 14 residues in the N-terminal domain of MHV S1 subunit [21]. Among these, residues I41, R20, and N26 of CEACAM1 and residues I22, Y162, Y162, and Y162 of MHV S protein are particularly important. Although closely related to MHV, *rat coronavirus* (RCoV) and *rat sialodacryoadenitis virus* (SDAV) do not use CEACAM1 as an entry receptor [22].

Several *alphacoronaviruses*, including *feline coronavirus* (FCoV), *canine coronavirus* (CCoV), porcine CoVs (TGEV and *porcine epidemic diarrhea virus* (PEDV)), and HCoV-229E, use aminopeptidase N (APN; also known as CD13) of their own host species as receptors [57,58]. For example, human APN (hAPN) can only be used by HCoV-229E; porcine APN can only be used by TGEV and PEDV; and canine APN can only be used by CCoV. Intriguingly, feline APN can be used by HCoV-229E, FCoV, CCoV, and TGEV [59]. APN is expressed as heavily glycosylated dimers in a variety of cells including epithelial cells of the kidney, respiratory and enteric tracts, endothelial, macrophage, dendritic cell, and cells at synaptic junction site [60]. The high-level expression of APN on respiratory and enteric tracts may be important for virus infection, tissue tropism, and transmission.

Critical domains and residues for virus binding have been identified in the APNs. The introduction of a glycosylation site at residues 291–293 abolished the receptor activity for HCoV-229E [6]. For TGEV, a C-terminal domain, aa 717–813, of porcine APN is essential [61]. Additionally, a C-terminal domain (aa 643–841) of canine APN in the hAPN backbone can mediate entry of CCoV, TGEV, and FCoV [62]. In feline APN, the N-terminal aa 135–295 is required for HCoV-229E, while the C-terminal aa 670–840 is required for FCoV, TGEV, and CCoV [63]. Further characterization mapped the key determinant to aa 288–290 for HCoV-229E, aa 732–746 for TGEV, and both aa 732–746 and 764–788 for FCoV and CCoV. The importance of these determinants was verified by introducing into mouse APN that is not the receptor for these *alphacoronaviruses*. Introduction of aa 732–746 of feline APN into mouse APN conferred the ability to support TGEV entry, while the introduction of both aa 732–746 and 764–788 conferred receptor ability for FCoV and CCoV [64]. Therefore, the N-terminal domain of APNs is recognized by human CoV, while the C-terminal domain is recognized by animal CoVs. Tusell and Holmes (2007) also showed that residue T742 of feline APN plays a critical role in receptor function [64]. Substitution of threonine with valine at this residue abolished the receptor activity for FCoV, CCoV, and TGEV, whereas a serine substitution at this residue was tolerable, underscoring a role of the hydroxyl group of T742 in the interaction with S proteins. Similarly, a feline APN mutant with substitution of T742 by the hAPN counterpart (R741) no longer supported FCoV, CCoV, and TGEV entry. Conversely, an hAPN mutant with R741T substitution gained receptor activity for TGEV but not for FCoV and CCoV [64].

Surprisingly, HCoV-NL63, an *alphacoronavirus* that is closely related to HCoV-229E, does not use hAPN as a receptor. Rather, it shares the same receptor, human angiotensin-converting enzyme 2 (ACE2), with two deadly human *betacoronaviruses*, *severe acute respiratory syndrome coronavirus* (SARS-CoV) and *severe acute respiratory syndrome coronavirus* 2 (SARS-CoV-2) [65,66,67]. The receptor activity of animal ACE2 orthologs has also been examined. Both SARS-CoV and SARS-CoV-2 are able to use ACE2 from many other animal species (e.g., palm civet, raccoon dog, dog, cat, etc.) as a receptor [68,69,70,71,72,73]. Some SARS-like bat coronaviruses, e.g., WIV1, are also able to use bat ACE2 and multiple animal ACE2 orthologs as a receptor [74,75], highlighting the possibility that these bat CoVs may be able to jump to humans. Another deadly human *betacoronavirus*, *middle east respiratory syndrome coronavirus* (MERS-CoV), uses dipeptidyl peptidase 4 (DPP4, also known as CD26) as a functional receptor [76]. Interestingly, APN, ACE2, and DPP4 are all peptidases.

### 3.2. Receptor-Binding Domains and Residues in the Coronaviral S Proteins

Receptor binding is primarily mediated by an independently folded domain, called receptor-binding domain (RBD), within the S1 domain of S protein. RBDs have been mapped to aa 407–547 for HCoV-229E [32,33], aa 506–655 for TGEV [23], aa 1–330 for MHV [38], aa 318–510 for SARS [46,77], aa 358 to 588 for MERS-CoV [44], and aa 331–524 for SARS-CoV-2 [50] (Table 1). All these RBDs are located at the C-terminal domain of the S1 (S1-CTD), except for the MHV RBD that is located at the N-terminal domain of the S1 (S1-NTD).

Critical receptor-binding residues in the RBDs have been identified for several CoVs by mutagenesis analysis and crystal structure determination of receptor-RBD complex [21,58,78,79]. For example, residues 33, 62, 79, 82, 162, 183, 212, 214, and 216 in the MHV RBD are important for virus infection [21,80,81]. Unexpectedly, residue G29 was found to be critical for the conformational changes triggered by either receptor binding or high pH [82].

Based on the crystal structure of SARS-CoV RBD complexed with hACE2 [83], a total of 14 amino acids in the RBD interact with ACE2. Among these residues, N479 and T487 are critical for SARS-CoV to jump from the amplifying host civet to humans and contribute to the pathogenicity. Sequence analysis showed that all the severe human isolates have N479 and T487; all the mild human isolates have N479 and S487, while all the animal isolates have K479 and S487. Replacement of K479 and S487 in civet isolate with N479 and T487 significantly enhanced hACE2-binding affinity and virus entry [68].

### 3.3. Attachment Factors

#### 3.3.1. Sialic Acid (SA)

Sialic acid (SA) refers to a group of N- or O-substituted derivatives of neuraminic acid, a monosaccharide with a nine-carbon frame. They are normally conjugated with glycoproteins or glycolipids and are widely distributed in animal tissues. While some studies have claimed that SA functions as a receptor for HCoV-OC43 and BCoV [84], a more recent paper demonstrated that the binding of the S protein to SA did not trigger a conformational change in HCoV-OC43 [85]. Therefore, we still consider SA to be an attachment factor in CoVs that enhances virus binding to host cells and contributes to entry.

SA-binding activity of the S protein has been implicated in the enterotropism of TGEV [24]. TGEV is able to replicate in both respiratory and enteric epithelial cells and causes severe diarrhea in newborn piglets [86]. The S protein can induce erythrocyte agglutination, which is mediated by the S-SA interaction. TGEV mutant, with seven mutations in the S1-NTD, was found to lose the hemagglutinating (HA) activity and enteropathogenicity [12,87], suggesting a link between SA-binding ability and the enteropathogenicity. Interestingly, a naturally occurring TGEV variant, *porcine respiratory coronavirus* (PRCoV), with N-terminal 227 aa deletion in the S protein, also loses the HA activity and only causes moderate respiratory tract infection [24]. Since PRCoV and other HA-deficient mutants can still replicate in cultured cells, it seems that SA-binding activity is important only in vivo. Based on the finding that wt TGEV, but not HA-deficient mutant, could bind to a carbohydrate-rich mucin-like sialoglycoprotein (MGP), which is abundantly expressed in jejunal goblet cells [88,89], it was proposed that SA-binding activity may assist TGEV in access to its target cells. It should be noted that porcine APN-expressing enterocytes in the intestine are covered by thick layers of mucus and glycocalix that are rich in carbohydrates, including SA. Binding to MGP via SA may allow TGEV to stay longer in the intestine and more easily cross the layers to initiate infection in enterocytes [88,89].

*Bovine coronavirus* (BCoV) and HCoV-OC43 are closely related *betacoronaviruses* [39]. Both the S proteins exhibit HA activity and can use N-acetyl-9-O-sialic acid (Neu5,9Ac2) as an attachment factor [41,43]. Removal of SAs from the cell surface inhibits virus infection in cultured cells, while resialylation restores infection. Therefore, the role of SA-binding activity in infection is more important in BCoV/HCoV-OC43 infection than in TGEV infection. However, there is a subtle difference in recognizing SA by these two CoVs. BCoV is more efficient in recognizing α2,3-linked form, whereas HCoV-OC43 prefers the α2,6-linkage [90]. It is unclear if this moiety must be linked to a specific glycoprotein or glycolipid or if there is a specific protein receptor for these two viruses. It has been reported that HCoV-OC43 can bind to a major histocompatibility complex class I molecule [91], but its receptor function has never been established.

SA also plays an important role in IBV infection. IBV S protein also has HA activity and preferentially binds to α2,3-linked SA. Removal of SAs from the surface of Vero, hamster kidney, chicken embryonic kidney cells, and trachea epithelial cells with neuraminidase reduced their susceptibility to IBV infection. Therefore, SA might serve as the primary binding determinant for IBV to facilitate the binding to its authentic protein receptor, though tighter binding and subsequent membrane fusion may require interaction with a second receptor [54,92].

In contrast to most of the RBDs that are located at the C-terminal part of the S1 domain (S1-CTD), the N-terminal part of the S1 (S1-NTD) has been known to bind to SA, which is mediated by a sugar-binding structure similar to human galectin in the S1-NTD [21]. Interestingly, the S1-NTDs of IBV, BCoV, HCoV-OC43, TGEV, and MERS-CoV, but not those of MHV, HCoV-HKU1, and SARS-CoV, possess the sugar-binding ability [21,78,93]. This difference was found to be due to the different lengths of a loop in S1-NTDs among these CoVs [21].

#### 3.3.2. Heparan Sulfate (HS)

Heparan sulfate (HS) is a common type of glycosaminoglycans (GAGs), a group of long unbranched polysaccharides that are composed of repeating disaccharide units. GAGs are attached to serine residues on proteoglycans during posttranslational modifications in the Golgi apparatus [94]. HS is found in the surfaces of most mammalian cell types and extracellular matrices.

In coronaviruses, using HS as an attachment factor is often a result of cell culture adaptation. The S protein of FCoV-UCD, a field isolate that does not grow in cell culture, is cleaved at a furin-recognition site, RRSRR, but cannot bind to heparin, an analog of cellular HS. On the contrary, a cell culture-adapted strain, UCD1, has an R-to-G substitution at the furin motif (RRSRG), which otherwise creates a heparin-binding motif (SRRSRG). Consistent with these genetic changes, the S protein is not cleaved but can bind to heparin. Meanwhile, the entry of UCD1 into FCWF cells became HS-dependent [35]. Similarly, persistent infection of MHV-A59 in murine cell culture resulted in an emergence of variant, MHV-BHK, which extended its host range by acquiring the ability to use HS for cell entry due to the acquisition of two HS-binding sites in its S protein [95,96]. Additionally, the S protein of this variant could no longer be cleaved, although the furin-cleavage motif is still there [95]. Together, these data suggest that acquisition of HS-binding ability is traded off for the loss of furin cleavage during cell culture adaptation. Unlike MHV-BHK, a neurotropic strain of MHV, JHMV, only binds to HS on the cell surface but does not use it as a receptor [97]. HS has also been shown to assist in SARS-CoV and SARS-CoV-2 entry by binding to the S protein directly, facilitating cell attachment and entry [47]. In *gammacoronaviruses*, HS has been considered to act as an attachment factor to mediate infection of IBV-Beaudette, an embryo-adapted strain that has extended the host range [55].

#### 3.3.3. C-Type Lectins

DC-SIGN (dendritic cell-specific ICAM-3-grabbing nonintegrin) and its homolog L-SIGN (for liver/lymph node-specific; also called CD209L or DC-SIGNR) are type II C-type lectins. DC/L-SIGN mediate cell adhesion and pathogen recognition. They have been implicated in the entry, infection, and transmission of many different viruses [98,99,100,101]. On one hand, they enhance virus entry and facilitate infection in cells expressing the cognate receptor. This is called cis-infection. On the other hand, they capture and transfer virus particles to target cells. This is called trans-infection [100,102,103]. DC-SIGN has been found to reside within a well-defined cholesterol-rich microdomain, called lipid raft, on the plasma membrane, which may act as a docking site for viruses to invade host cells [104].

The role of DC/L-SIGN in SARS-CoV entry is controversial. It has been shown that DC-SIGN and/or L-SIGN could serve as an attachment factor to augment the entry of SARS-CoV [48,49], HCoV-NL63 [31], and FIPV [36]. DC/L-SIGN has also been confirmed to promote viral infection in IBV, TGEV, and the newly emerged SARS-CoV-2 [25,51,56]. However, some studies showed that DC/L-SIGN are alternative and independent albeit less efficient receptors for HCoV-229E [34] and SARS-CoV [105,106]. Interaction with DC/L-SIGN requires at least seven glycosylation sites (N109, N118, N119, N158, N227, N589, and N699) in the SARS-CoV S protein, which is outside of the ACE2-binding domain (aa 318-510) [105].

L-SECtin, another C-type lectin co-expressed with L-SIGN on sinusoidal endothelial cells in liver and lymph nodes also promotes SARS entry, but not through binding to high-mannose glycan as DC/L-SIGN do [107].

## 4. Membrane Fusion

### 4.1. Structure and Function of the S2 Domain

Membrane fusion is mediated by the S2 domain of coronaviral S proteins. The S2 domain contains several structurally conserved regions: fusion peptide (FP), two heptad repeats (HRs), TM domain, and cytoplasmic tail (Figure 2), all of which contribute to membrane fusion. The fusion peptide is highly hydrophobic and is able to cause leakage of liposomes [108]. Mutational analysis confirms that fusion peptide is important for membrane fusion [109,110,111].

The most striking feature of the S2 domain is two HRs. HR1 is located in the N-terminus, right downstream of the fusion peptide, and HR2 is located in the C-terminus, upstream of the TM domain. HR is a motif with seven-residue periodicity. The first and fourth residues are typically hydrophobic, whereas the others are hydrophilic. This motif is the basis of the α-helix coiled-coil structure and plays a central role in membrane fusion [112].

**Figure 3:**
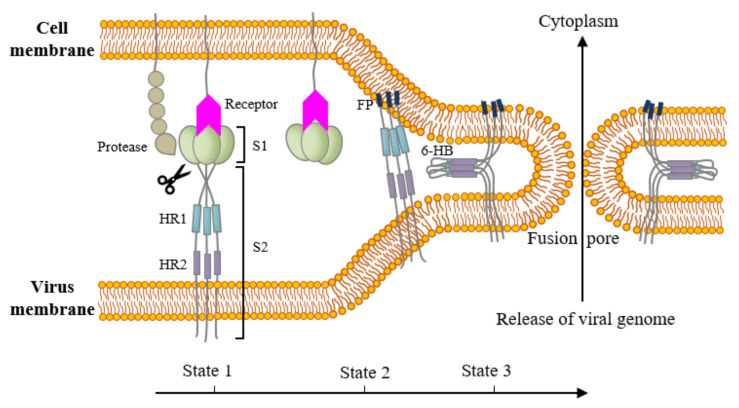
Schematic model for membrane fusion between coronavirus particles and host cells. State 1 (native): the S1 domain is associated with the S2 domain. The fusion peptide (FP) is buried inside the structure and both of the heptad repeats (HRs) exist as trimers individually. State 2 (intermediate state): the S1 domain is dissociated from the S2 domain after receptor binding. Proteolytic cleavage at the S1/S2 boundary and/or at the S2′ site helps the FP expose and then insert into the cell membrane. State 3 (fusion active state): HR2 folds back to HR1 and forms a stable six-helix bundle, which brings viral and cellular membranes into close proximity, forming a fusion pore, which is followed by releasing viral genome into the cytoplasm of host cells. FP: fusion peptide. HR1: heptad repeat 1. HR2: heptad repeat 2. 6-HB: 6-helix bundle.

Based on the current model of membrane fusion of class I fusion proteins (Figure 3) [113], the S protein has three conformational states. Under native, pre-fusion state (state 1), the S protein is metastable, in which the S1 is associated with the S2. During this period, the fusion peptide is not exposed, and two HRs form trimeric helix coiled-coil individually. Following receptor binding, the S1 sheds from the S2. Subsequently, primed by the proteolytic cleavage at the S1/S2 boundary and/or at the S2′ site, the fusion peptide exposes and is inserted into the cell membrane. This state is known as the intermediate state (state 2). At state 3, HR2 folds back to HR1 and together forms a six-helix bundle, in which HR1 trimeric coiled-coil lies in the inner core that is wrapped by an outer layer of antiparallel HR2 coiled-coil [109,112,113,114,115]. The association of HR1 and HR2 represents the postfusion state. This six-helix bundle brings the fusion peptide, TM domain, and cell membrane into close proximity, destabilizing the lipid bilayers, forming a fusion pore, and releasing the viral genome into the cytoplasm [113,116,117]. HRs are one of the major targets for antiviral therapy. HR peptides or HR-specific antibodies can efficiently inhibit CoVs infection by blocking the formation of six-helix bundles [109,118,119,120].

Other regions in the S2 domain are also involved in membrane fusion. The region between two HRs is called the interhelica (IH) domain. It has been shown in MHV and SARS-CoV that a hydrophobic region of the IH domain is important for cell-to-cell fusion [110,111]. A short region immediately upstream of the TM domain, called juxtamembrane domain (JMD), is rich in aromatic residues (KWPWYVWL) and is highly conserved in CoVs. Alanine substitution of the aromatic residues results in reduced virus-to-cell fusion and cell-to-cell fusion [121].

The TM domain is highly conserved with up to 30% amino acid identity among different CoVs. Studies with SARS-CoV and MHV show that the TM domains contribute to the stability of S trimers and membrane fusion [122,123,124].

The cytoplasmic tail contains conserved sequences that are important for S protein trafficking through the cellular secretory pathway, virus particle assembly, and membrane fusion [125]. An ER-targeting signal is located at the very end of cytoplasmic tail. In IBV, the signal is a dilysine motif (KKSV); mutations of the residues KK cause faster trafficking of IBV S proteins to the cell surface, virus growth defect, and premature syncytia formation [126]. The S proteins of *alphacoronaviruses* and IBV also contain a tyrosine-based endocytic signal (YXXθ, where θ represents a bulky hydrophobic residue) upstream of the ER-targeting signal. This motif binds to the AP2 adaptor complex that, in turn, binds to clathrin to induce endocytosis [127]. Youn et al. (2005) demonstrated that this signal rapidly endocytosed IBV S proteins from the plasma membrane [126]. Deletion of such an ER-targeting signal and endocytic signal significantly enhanced cell entry of TGEV [128], HCoV-NL63 [129], SARS-CoV [130], and SARS-CoV-2 [73]. In the N-terminal and central regions of the cytoplasmic tail, there are four conserved cysteine clusters. These cysteines are important for membrane fusion. In particular, the clusters I and II cysteines near the TM domain play a major role [122,124,131]. These two cysteine clusters are also the major sites for palmitoylation in SARS-CoV S protein [131]. Palmitoylation in MHV S is critical for cell-to-cell fusion, assembly, and infectivity [132].

### 4.2. Cleavage of S Proteins by Host Proteases

Proteolytic cleavage of the S proteins is a critical priming step for membrane fusion mediated by class I fusion proteins. Coronaviral S proteins can be cleaved at different sites. Two main types of protease cleavage sites, S1/S2 and S2′, have been identified. The former is located at the S1/S2 boundary; the latter is located right upstream of the fusion peptide (Figure 2) [5]. Depending on the cell type, S proteins are cleaved at different stages of the virus life cycle and at different cellular sites, e.g., during the S protein biogenesis at the ER/Golgi and trans-Golgi compartment, on the plasm membrane or in endosome/lysosome during cell entry, and in the extracellular space during infection. This spatially and temporally controlled and cell-type-dependent cleavage modulates the pathogenicity, cell and tissue tropism, and host range of coronaviruses.

A number of host proteases have been identified to activate membrane fusion of CoVs. These include trypsin, furin, cathepsins, TMPRSS, elastase, etc. [5]. Among these, furin, trypsin, cathepsin L and TMPRSS could cleave at the S1/S2 site, while trypsin and elastase could cleave at the S2ʹ site. In general, furin cleavage occurs during biosynthesis; trypsin and TMPRSS cleavage occur in the extracellular space and plasm membrane; cathepsin cleavage occurs in endosome/lysosome (Figure 1).

For some CoVs, e.g., IBV, MHV, CCoV, and FCoV-I, the S proteins have a multibasic furin cleavage motif (often RRXRR) at the S1/S2 boundary. Thus, these S proteins are cleaved during biosynthesis by furin-like protease into S1 and S2 subunits prior to assembly into virion [35,133]. Furin cleavage is not essential for virus–cell-membrane fusion, but it enhances cell-to-cell fusion [122,134,135,136]. Persistent infection of cell culture often leads to loss of furin cleavage site, and such cell-adapted variants are generally attenuated in vivo [134]. For MHV-A59 strain, the S protein is cleaved a second time at the S2ʹ site during virus entry [137]. The S protein of IBV is also cleaved at the S2ʹ site during protein biosynthesis and during infection, and this cleavage has been shown to be important for virus entry [138].

For those CoVs that do not have furin-cleavage sites, e.g., PEDV and FCoV-II, other proteases, including TMPRSS2, trypsin, or cathepsin, are used. For example, PEDV S protein was found to be cleaved at the S2ʹ site by trypsin after receptor binding [139], and the virus entry was shown to be dependent on a low pH and endosomal cathepsin [140]. Cleavage at the S2ʹ site by cathepsin was also found to be critical for FCoV-II entry [141]. Proteolytic cleavage by TMPRSS2 has been found to promote entry at the plasm membrane for SARS-CoV [142], MERS-CoV [143], and SARS-CoV-2 [144]. In addition to TMPRSS2, SARS-CoV also uses other proteases, e.g., cathepsin L, trypsin, thermolysin, elastase, and factor Xa, to cleave the S protein and promote entry [145,146,147].

Gain or loss of proteolytic cleavage site is often associated with changes in the entry route, dependence on pH, and cell-to-cell fusion ability. HCoV-229E S protein does not have a furin cleavage site but is cleaved by cathepsin L and maybe other proteases. This cleavage is required for membrane fusion in acidic endosomes. However, if the virus is treated with trypsin before infection, the S protein will be cleaved, coupled with cell surface entry under neutral pH and enhanced syncitia formation [148]. This phenomenon is also observed in SARS-CoV infection with trypsin treatment; furthermore, the introduction of furin cleavage site into SARS-CoV S results in a cleaved S that mediates entry at the cell surface in a pH-independent fashion [146,149]. Similarly, MHV-A59 expresses a cleavable S protein that mediates entry at the cell surface and cell-to-cell fusion under neutral pH. In contrast, MHV-2 expresses an uncleaved S protein that mediates cathepsin-dependent entry in the acidic endosome and cannot mediate cell-to-cell fusion. Recombinant virus bearing a cleaved MHV-2 S protein has an A59-like phenotype in entry [150]. These findings suggest that, during in vivo infection, HCoV-229E, SARS-CoV, and MHV-2 may bypass the endocytosis pathway and directly enter target cells from the cell surface, since their S proteins may be cleaved by various proteases secreted extracellularly.

## 5. Host Restriction Factors for Coronavirus Entry

### 5.1. IFITM

Interferon-induced transmembrane proteins (IFITMs), more specifically, IFITM1, -2, or -3, have been shown to inhibit cell entry of many different enveloped RNA viruses [151,152,153]. Several HCoVs, including SARS-CoV [154], SARS-CoV-2 [155], 229E [156], and MERS-CoV [157], have been found to be sensitive to IFITMs restriction.

How IFITMs inhibit virus entry is not completely understood. The current model proposes that IFITMs inhibit virus-cell fusion at the hemifusion or pore formation stage by modifying the rigidity and/or curvature of the membranes in which they reside [158]. IFITM1 mostly resides at the plasma membrane, while IFITM2 and -3 are more found in endosome/lysosomal. A compelling model proposes that IFITM2/3 block virus entry at endosome/lysosome by interrupting the membrane fusion [151]. The following evidence supports this model: (i) IFITMs-insensitive viruses fuse at the plasma membrane; (ii) mutant IFITM3 that could not be internalized into endosome/lysosome had impaired restriction activity against influenza virus; (iii) restriction of SARS-CoV, SARS-CoV-2, and bat CoV WIV1 by IFITMs could be circumvented by pre-treatment with trypsin or TMPRSS2 that bypasses the dependence on lysosomal cathepsin L [75,154,159]; and (iv) removing the furin cleavage site in the S protein of SARS-CoV-2 made the S proteins remain noncleaved during biosynthesis and the entry more dependent on low pH and endosome/lysosome, and as a result, the entry is more sensitive to IFITM2 restriction [155].

IFITMs do not always inhibit viral entry. HCoV-OC43 could use IFITM2 or IFITM3 to promote its cell entry and infection, presumably by modulating membrane fusion [153,160]. It remains largely unknown how IFITMs promote CoV entry. Recent studies have indicated that specific mutations can switch the biological activity of IFITMs from inhibiting to enhancing the infection of selected human coronaviruses [161]. Three distinct mutations in IFITM1 or IFITM3 were found to allow the host restriction factors to promote viral entry in SARS-CoV and/or MERS-CoV. For example, an alanine or aspartic acid replacement of IFITM3 tyrosine 20 to mimic unphosphorylated or phosphorylated IFITM3 reduced its inhibition activity on HCoV-NL63 and -229E entry but enhanced the entry of SARS-CoV and MERS-CoV. These findings suggest that the residues and structural motifs of IFITMs are vital for modulating HCoV entries, likely through viral/host cellular components’ interactions at the site of entry to modulate membrane fusion [161].

### 5.2. LY6E

Lymphocyte antigen 6 family member E (LY6E) potently restricts infection by multiple CoVs, including MHV and several HCoVs (229E, OC43, MERS-CoV, SARS-CoV, SARS-CoV-2) [160,162]. Mechanistic studies revealed that LY6E inhibits coronavirus entry into cells by interfering with S protein-mediated membrane fusion [162]. Mice lacking LY6E in immune cells were highly susceptible to mouse hepatitis virus (MHV). In LY6E knockout mice, an aggravated viral pathogenesis was accompanied by loss of hepatic immune cells, higher splenic viral burden, and reduction in global antiviral gene pathways. Furthermore, constitutive LY6E directly protects primary B cells from murine coronavirus infection [162]. While overexpression of amphotericin treatment or TMPRSS2 neutralized the restriction of IFITM3 on virus entry, LY6E restriction on human CoV entry was not affected [160]. These findings suggest that LY6E controls CoV infection via a mechanism distinct from IFITMs.

### 5.3. CD74

CD74 has been recently identified as a direct broad-range antiviral effector protein [163]. Among the four isoforms of CD74, only p41 isoform is able to block the endosomal entry pathway of EBOV and coronaviruses, including SARS-CoV, SARS-CoV-2, and Bat coronavirus WIV1 [163]. p41 is up-regulated by the major histocompatibility complex (MHC) class II transactivator (CIITA) but inhibits virus entry independently of CIITA expression. The p41 isoform contains the thyroglobulin domain, lacks the ER retention signal, and normally accumulates in endosomes. The CD74 thyroglobulin domain inhibits cathepsins, suggesting that this may be the mechanism for antiviral activity. Disruption of the p41 CTSL binding site by mutation completely inhibited antiviral activity.

### 5.4. Ezrin

Ezrin, a membrane actin-linker, was discovered as a CoV entry restrictor by using the cytoplasmic tail of SARS-CoV S protein as bait in a yeast two-hybrid screen [164]. Further characterization showed that the F1 lobe of the FERM domain of Ezrin interacts with both the eight C-terminal residues and the conserved cysteine cluster near the TM domain. Mechanistic study implies that Ezrin inhibits SARS-CoV entry possibly by restricting the fusion pore opening and trapping incoming particles within the intracellular filamentous actin network [164].

### 5.5. GILT

Recently, we found that gamma-interferon-inducible lysosomal thiol reductase (GILT) could inhibit pseudotyped virus entry of multiple enveloped RNA viruses, including SARS-CoV [165]. GILT is constitutively expressed in lung epithelial cells and fibroblasts. Its expression could be further induced by type II interferon. We also found that GILT expression reduced the level and activity of endosome/lysosome-associated cathepsin L, while trypsin treatment could abrogate the entry restriction by GILT. These findings suggest that GILT exerts its restriction in the endosome/lysosome.

### 5.6. CH25H

Cholesterol 25-hydroxylase (CH25H) is also an interferon (IFN)-stimulated gene. The enzyme converts cholesterol to 25-hydrocholesterol (25HC) that has been known to possess broad antiviral activities against a wide range of enveloped viruses by blocking membrane fusion. Most recently, 25HC was shown to accumulate in the late endosomes and inhibit SARS-CoV-2 infection [166,167]. Mechanistically, this inhibitor may be achieved by activating the ER-localized acyl-CoA:cholesterol acyltransferase (ACAT) that depletes cholesterol from the plasma membrane, which leads to interruption of membrane fusion.

## 6. Perspective of Future Study

Coronavirus research has been significantly stimulated twice over the past two decades. The first was triggered by the outbreak of SARS-CoV during 2002-2003 that caused 774 deaths in 8096 infected people in the world (http://www.who.int/csr/sars/country/table2004_04_21/en/index.html, accessed on 9 September 2021). The second was initiated by the global pandemic of SARS-CoV-2 since December 2019. As of 30 August 2021, this virus has caused ~220 million confirmed cases and nearly 4.5 million deaths (https://COVID19.who.int/, accessed on 9 September 2021). As a result, numerous efforts have been made, and accordingly, significant progress has been made in every direction of CoV research. The most striking finding in the field of CoV entry over the past ten years is probably the identification of a number of host restriction factors. These factors share several common features. First, they are all interferon-stimulated genes (ISGs). Second, they are against a broad range of RNA viruses. Third, they work by inhibiting the membrane fusion during virus entry. Despite this progress, we still have very limited information as to how these restriction factors exert their inhibitor effect mechanistically. Additionally, there are hundreds of ISGs. It was estimated that nearly 10% of human genes are able to be stimulated by interferons [168]. We speculate that there will be more ISGs that are able to inhibit CoVs entry. This field warrants more studies.

Another important field to pursue in the future is the identification of host factors, including receptors, attachment factors, and other host proteins, that promote cell entry of CoVs. So far, 45 CoV species have been recognized, but only a handful of them have receptors identified (Table 1). Receptors of several important animal CoVs, e.g., IBV, BCoV, and *swine acute diarrhea syndrome coronavirus* (SADS-CoV), remain unknown. SADS-CoV is a novel emerging virus that originated from a bat coronavirus by cross-species transmission. It caused a large-scale outbreak of fatal disease in pigs, resulting in 24,693 piglet deaths across four farms in China [169]. Initial studies showed that several known receptors for CoVs, including ACE2, APN, and DPP4, are not the receptor for SADS-CoV [169,170].

Significant progress has also been made in understanding the S-receptor molecular interaction by determining the crystal structures of their complex. However, such information is still lacking for many important CoVs and thus further investigation is needed.

## Figures and Tables

**Table 1:** Host receptor usage, receptor binding domains, and attachment factors for CoVs.

Genera	Species *	Receptor	Receptor Binding Domain (RBD)	Attachment Factor
*Alphacoronaviruses*	TGEV	Porcine APN	C-terminal aa 506–655 [23]	Sialic acid [24], DC/L-SIGN [25]
	PEDV	Porcine APN	C-terminal [26]	Sialic acid [27], heparan sulfate [28], DC/L-SIGN [25]
	HCoV-NL63	ACE2	C-terminal aa 476–616 [29]	Heparan sulfate [30], DC/L-SIGN [31]
	HCoV-229E	Human APN (also called CD13)	C-terminal aa 407–547 [32,33]	DC/L-SIGN [34]
	FCoV	Feline APN	Unknown	Heparan sulfate [35], DC/L-SIGN [36]
	CCoV	Canine APN	C-terminal aa 526–676 [37]	
*Betacoronavirus*	MHV	CEACAM1 (also called CD66a)	N-terminal aa 1–330 [38]	Sialic acid [27], Heparan sulfate [39]
	BCoV	Unknown	Not yet determined but falls within the N-terminal domain [40]	Sialic acid [41], heparan sulfate [39]
	HCoV-OC43	Unknown	aa 339–549 [42]	Sialic acid [43], heparan sulfate [35]
	MERS-CoV	DPP4 (also called CD26)	aa 358–588 [44]	Sialic acid [27], heparan sulfate [45]
	SARS-CoV	ACE2	aa 318–510 [46]	Heparan sulfate [47], DC/L-SIGN [48,49]
	SARS-CoV-2	ACE2	aa 331–524 [50]	Heparan sulfate [47], DC/L-SIGN [51]
	HCoV-HKU1	Unknown	N/A	O-Acetylated Sialic Acid [52]
*Gammacoronavirus*	IBV	Unknown	N-terminal residues 19–272 [53]	Sialic acid [54], Heparan sulfate [55], DC/L-SIGN [56]

* TGEV: *porcine transmissible gastroenteritis virus*; PEDV: *porcine epidemic diarrhea virus*; HCoV-NL63: *human coronavirus NL63*; HCoV-229E: *human coronavirus 229E*; FCoV: *feline coronavirus*; CCoV: *canine coronavirus*; MHV: *mouse hepatitis virus*; BCoV: *bovine coronavirus*; HCoV-OC43: *human coronavirus OC43*; MERS-CoV: *middle east respiratory syndrome coronavirus*; SARS-CoV: *severe acute respiratory syndrome coronavirus*; SARS-CoV-2: *severe acute respiratory syndrome coronavirus 2*; HCoV-HKU1: *human coronavirus HKU1*; IBV: *infectious bronchitis virus*. The references are provided in the bracket for the RBDs and attachment factors.
